# LKB1 Loss Assessed by Immunohistochemistry as a Prognostic Marker to First-Line Therapy in Advanced Non-Small-Cell Lung Cancer

**DOI:** 10.3390/curroncol30010027

**Published:** 2022-12-26

**Authors:** Alejandro Avilés-Salas, Diego A. Díaz-García, Luis Lara-Mejía, Andrés F. Cardona, Mario Orozco-Morales, Rodrigo Catalán, Norma Y. Hernández-Pedro, Eduardo Rios-Garcia, Maritza Ramos-Ramírez, Oscar Arrieta

**Affiliations:** 1 Thoracic Oncology Unit, Instituto Nacional de Cancerología (INCan), Mexico City 14080, Mexico; 2 Personalized Medicine Laboratory, Instituto Nacional de Cancerología (INCan), Mexico City 14080, Mexico; 3 Direction of Research and Education, Luis Carlos Sarmeinto Angulo Cancer Treatment and Research Center—CTIC, Bogotá 110131, Colombia; 4 Neuroimmunology Laboratory, Instituto Nacional de Neurología y Neurocirugía, Mexico City 14269, Mexico

**Keywords:** lung cancer, advanced NSCLC, STK11, prognostic biomarker, immunohistochemistry

## Abstract

(1) Background: Liver kinase B1 (LKB1) is a tumor suppressor gene involved in cell growth and metabolism. However, its alterations are not routinely assessed for guiding therapy in clinical practice. We assessed LKB1 expression by immunohistochemistry as a potential biomarker. (2) Methods: This bicentric retrospective cohort study analyzed data from patients with advanced NSCLC who initiated platinum-based chemotherapy or epidermal growth factor receptor- tyrosine kinase inhibitor (EGFR-TKI) between January 2016 and December 2020. Kaplan–Meier and Cox regression models were used for survival curves and multivariate analysis. (3) Results: 110 patients were evaluated, and the clinical stage IV predominated the lung adenocarcinoma histology. LKB1 loss was observed in 66.3% of cases. LKB1 loss was associated with non-smokers, the absence of wood smoke exposure and an EGFR wild-type status. The median progression-free survival (PFS) and overall survival (OS) in the population were 11.1 and 26.8 months, respectively, in the loss group, compared with cases exhibiting a positive expression. After an adjustment by age, smoking status, Eastern Cooperative Oncology Group Performance Score (ECOG-PS), EGFR status and type of administered therapy, LKB1 loss was significantly associated with worse PFS and OS. (4) Conclusion: Patients with an LKB1 loss had worse clinical outcomes. This study warrants prospective assessments to confirm the prognostic role of the LKB1 expression in advanced NSCLC.

## 1. Introduction

The liver kinase B1 (LKB1 or STK11) gene is in the short arm of chromosome 19 (19p13.3), and encodes a serine threonine kinase with a stability role in the regulation of cellular metabolism and energy homeostasis, including hypoxia and glucose deprivation, maintaining cell homeostasis and survival. The germline loss of function of this protein kinase is responsible for the Peutz–Jeghers syndrome (PJS), a genetic disorder associated with hamartomatous polyps and dark-colored spots. On the contrary, somatic inactivation of LKB1 increases the incidence of several cancers, including gastrointestinal, pancreatic and lung carcinomas [1].

LKB1 is a key serine/threonine kinase that acts as a cellular energy regulator. In low-energy conditions, LKB1 phosphorylates AMPK. The activation of this kinase inhibits anabolic pathways for more efficient production of ATP [2,3,4]. LKB1 also works as a tumor suppressor through AMPK activation. AMPK directly phosphorylates and inhibits the mammalian target of rapamycin (mTOR); in turn, its inhibition downregulates angiogenesis-related factors such as hypoxia-inducible factor 1 (HIF-1) and vascular endothelial growth factor (VEGF), thereby negatively affecting anaerobic glycolysis (Warburg effect) [2,5,6]. In addition to these functions, recent studies suggest that LKB1 may regulate the tumor microenvironment through AMPK-related kinases SIK1, SIK3, and STING, thereby affecting treatment response to immune checkpoint inhibitors [7,8].

Approximately 45.6% of Hispanics with metastatic non-small-cell lung cancer (NSCLC) harbor somatic *STK11* or *KEAP1* gene mutations without *KRAS* co-mutations or any other alterations, and around 15% had other concurrent mutations with *KEAP1* or *KRAS*, supporting the potential key role of LKB1 as a contributor to lung cancer genesis in sporadic tumors [9]. Ghaffar et al. reported that in lung atypical adenomatous hyperplasia, the loss of LKB1 expression (assessed by immunohistochemistry [IHC]) was strongly associated with severe dysplasia, suggesting that inactivation of LKB1 may play a role in the transition from premalignant to malignant transformation [10]. In this regard, LKB1 expression has been associated with genomic alterations with a prognostic and potentially predictive role in NSCLC patients [11]. Similarly, approximately 30% of breast cancer sporadic cancers were associated with low STK11 levels [12]. 

Genomic alterations in lung cancer have shown wide variability according to patient ethnicity and region [13,14], and particularly for LKB1 alterations, its prevalence determined by an IHC technique is unknown in Hispanics. Currently, most *STK11* alterations are mainly detected using next-generation sequencing (NGS) in tissue and liquid biopsies, representing a high economic burden for most economies in our region [15,16]. However, LKB1 antibodies employed to evaluate protein expression are a reasonable and less expensive alternative. Furthermore, evaluating LKB1 by IHC has been validated in the setting of lung cancer [11,17]. Even though LKB1 (STK11) is not routinely tested in the clinical setting, many studies have suggested a prognostic role in NSCLC [11]. This study aimed to analyze LKB1 expression in Hispanic patients with advanced NSCLC; it associated a negative expression with main clinicopathological variables and determined its prognostic role as a prognostic marker.

## 2. Materials and Methods

### 2.1. Patients

This retrospective cohort study analyzed data from de-identified electronic medical records of patients with an advanced (unresectable or metastatic) NSCLC diagnosis who were treated at two cancer centers in Latin America (LATAM) in Mexico and Colombia between January 2016 and December 2020. Eligible patients had available tissue to determine the LKB1 expression by immunohistochemistry. The study was conducted in accordance with the Declaration of Helsinki and approved by the Institutional Review Board of the Instituto Nacional de Cancerología (INCAN/CI/0603/2016) for studies involving humans. The Ethics Committee of the Institution waived individual informed consent due to the nature of the study.

Electronic medical records were evaluated by a multidisciplinary team that included a medical oncologist and a molecular biologist. Relevant clinical and histopathological variables were analyzed; including age, gender, smoking history, woodsmoke exposure history, targetable molecular alterations (e.g., EGFR), tumor differentiation grade, histological subtype and programmed death-ligand 1 (PD-L1) tumor proportion score (TPS; percentage of viable tumor cells showing partial or complete membrane staining on IHC assay). Patients were excluded if there was insufficient tissue to assess the LKB1 expression by IHC or if >20% of predetermined clinical or histopathological variables were unavailable. Individual patient information remained confidential during the entire protocol, and clinical decisions were not influenced based on the results of the present study.

### 2.2. Immunohistochemistry Stain

Immunohistochemistry stain was performed using a polyclonal commercial antibody (HPA017254 (Sigma-Aldrich)). After classic deparaffinization and rehydration, citrate buffer was used for antigen retrieval. Previously to incubation with secondary antibody, primary antibody was used to cover the samples for two hours. Chromogenic detection was performed with streptavidin-horseradish peroxidase conjugate (Universal LSAB kit, Dako Corp) and diaminobenzidine tetra-hydrochloride (liquid DAB, Dako Corp). Distilled water and hematoxylin (CatHE-M, Biocare Medical, CA, USA) were used for counterstaining. Finally, slides were mounted in Entellan medium (Merck & Co., NJ, USA). The entire procedure was performed by a highly experienced molecular biologist and expert pathologist.

### 2.3. Therapy

Patients without oncogenic driver (*EGFR*) receive platinum-based chemotherapy as upfront treatment. Those harboring an *EGFR* alteration receive a first- or second-generation tyrosine kinase inhibitor (TKI). Both therapies are delivered until disease progression or unacceptable adverse events. In further lines of therapy, patients without oncogenic drivers could receive immunotherapy at the discretion of the treating physician. Osimertinib was not available as second-line therapy in those patients who developed a T790M as a resistant mechanism after a previous EGFR-TKI.

### 2.4. Statistical Analysis 

For statistical analysis, categorical variables were reported as frequencies and proportions; comparisons among categorical variables were analyzed by the χ^2^ test or Fisher’s exact test. Continuous variables were reported as means and standard deviations (SD) or medians and interquartile ranges, based on data distribution. Comparisons of means were evaluated using a *t*-test or one-way ANOVA, while medians were compared using a Mann–Whitney *U* test. For survival curve analysis, all variables were dichotomized and analyzed in terms of progression-free survival (PFS) and overall survival (OS). PFS was calculated from the date of diagnosis to the date of progression or death for any cause. OS was measured from the date of diagnosis to death. Patients who did not develop the event at the last follow-up were censored at the last observation date. PFS and OS were analyzed by the Kaplan–Meier method, and the log-rank test assessed comparisons among subgroups. Patients who lost follow-up were censored from survival analysis. The multivariate analysis with a Cox proportional model estimates the hazard ratios, with 95% CI for disease progression and death. A *p*-value < 0.05 was predetermined to be considered statistically significant. All statistical analyses were performed using SPSS software, version 26 (SPSS Inc., Chicago, IL, USA).

## 3. Results

### 3.1. Patients

110 were enrolled; the median age was 60 ± 11.5 years, with a higher proportion of females (68.2%), and non-smokers (80.0%). Non-squamous NSCLC was diagnosed in 93.6%, the majority were moderately and poorly differentiated tumors (71.9%), and *EGFR* mutant tumors were observed in 27.3%. Clinical stage IV was the most common presentation (92%) at diagnosis, and most of the patients had a good Eastern Cooperative Oncology Group Performance Score (ECOG PS) (0–1) (91.8%). All baseline characteristics are summarized in Table 1.

**Figure 1:**
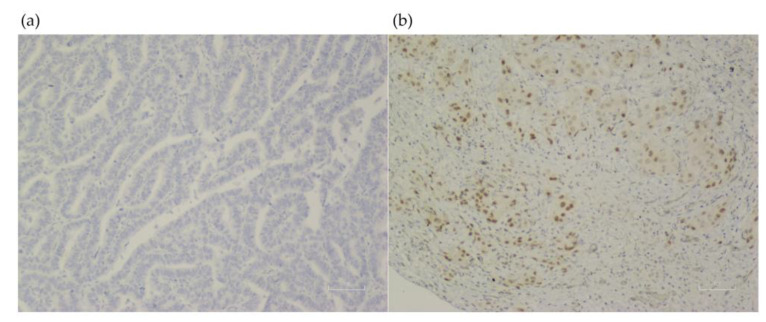
LKB1 expression assessment in lung tissue from samples of patients with lung adenocarcinoma (×100). (**a**) Negative LKB1 expression. (**b**) Positive LKB1 expression.

Clinical factors associated significantly with an LKB1 loss were never smokers (72.7% vs. 27.3%, *p* = 0.005), and those without wood smoke exposure (73.1% vs. 26.9%; *p* < 0.001). In the molecular analysis, the *EGFR* wild-type status also was associated with an LKB1 loss (77.5% vs. 22.5%; *p* < 0.001) Table 1. No other clinical, pathological, or molecular factors were associated with an LKB1 loss. LKB1 expression assessment by IHC is shown in Figure 1.

### 3.2. Progression-Free Survival According to LKB1 Status

**Figure 2:**
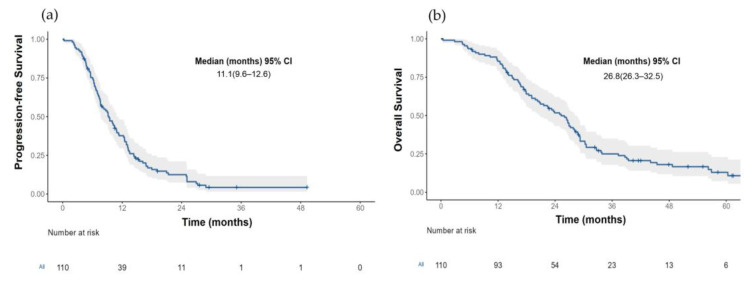
(**a**) Progression free survival and (**b**) Overall survival in the total population.

**Figure 3:**
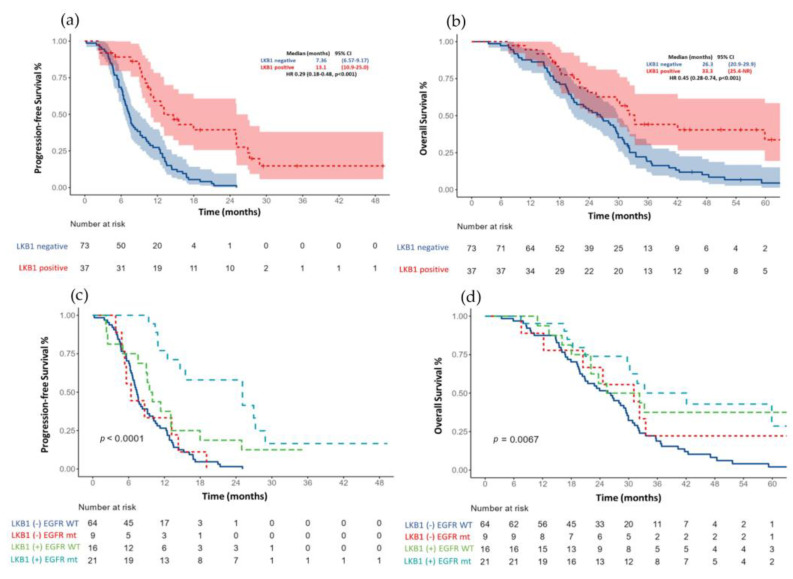
(**a**) Progression free survival based on LKB1 expression and (**c**) after adjustment for EGFR status. (**b**) Overall survival according to LKB1 expression and (**d**) after adjustment for EGFR status.

The median PFS for the entire cohort (*n* = 110) was 11.1 months (95% CI 9.61–12.6) Figure 2a. Those patients with an LKB1-positive expression had a more prolonged median PFS than patients with an LKB1 loss (13.1 months (95% CI, 6.6–8.5) vs. 7.36 (95% CI, 6.57–9.17); HR 0.29, *p* < 0.001) Figure 3a. 

After an adjustment for the *EGFR* status (wild-type versus mutant) and type of treatment (first- and second-generation EGFR-TKI vs. platinum-based chemotherapy). The median PFS continued to be significantly larger in those patients with a positive LKB1 expression, especially between *EGFR* mutant patients versus wild-type; Figure 3c. 

In the univariate analysis, the other factors associated with a worse PFS were the ECOG PS (2), clinical stage IV, *EGFR* wild-type status and the lack of response to first-line therapy. In the multivariate analysis, after the adjustment for ECOG PS, smoking status, tumor grade, clinical stage, *EGFR* status and type of response to first-line therapy, the LKB1-positive expression remained a significant factor for PFS (HR 0.20 (0.11–0.38)). Other associated factors that positively impact the PFS are detailed in Table 2.

### 3.3. Overall Survival according to LKB1 Status

The median OS for the total population (*n* = 110) was 29.4 months (95% CI 26.3–32.5) Figure 2b. Patients with an LKB1-positive expression had a median OS of 33.3 months (95% CI 25.4–NR) compared with 26.3 months (95% CI (20.9–29.9); HR 0.45 (0.28–0.75), *p* = 0.001) in those patients with an LKB1 loss; Figure 3b. After the adjustment for the EGFR status (wild-type versus mutant), the median OS continued to be significantly larger in those patients with a positive LKB1 expression, especially *EGFR* wild-type tumors, Figure 3d. In the univariate analysis for overall survival, age (≥60 years), ECOG PS (2), tumor grade (moderately, and poorly differentiated tumors), EGFR wild-type status and the absence of response were associated with a worse OS. After an adjustment for age, smoking status, *EGFR* status, ECOG PS and type of response to first line therapy, the LKB1-positive expression remained a significant factor for OS (HR 0.34 (0.18–0.65); *p* = 0.001); Table 3. 

## 4. Discussion

LKB1 is a tumor suppressor gene commonly altered in NSCLC patients (30–35%) [11,18,19]. Most of the somatic mutations in sporadic NSCLC within the LKB1 gene resulted in either a truncated or absent protein that provokes an inactive or malfunctioning product that favors an uncontrolled growth of tumor cells [20]. Although most of the current evidence has assessed the prognostic and potentially predictive role of LKB1 mutations with PCR or next-generation sequencing analysis, it still unknown whether the immunohistochemistry analysis will discriminate in the same way. This became relevant when underscoring the simplicity and cost effectiveness of immunohistochemistry for evaluating LKB1 expression [17].

In this retrospective cohort, we analyzed the LKB1 expression by IHC in 110 metastatic NSCLC patients. Unlike previous reports, the frequency of LKB1 loss of expression evaluated by IHC was much higher in our population (65.5%), which might be related to a wide variation in racial and ethnic differences. In the Asian population, LKB1 loss varies from 3–7%, whereas the range in Caucasians is much broader (17–42%) [21]. This is concordant with LKB1 mutations, which occurred more often in the US than Asian countries (17% vs. 5%; *p* = 0.001) [21].

In previous reports, clinicopathological and molecular characteristics have been associated with a negative LKB1 expression. In this regard, one metanalysis indicated that current and former smokers, poorly and moderately differentiated tumors and those tumors with a nodal involvement were associated with a low/negative LKB1 expression. Moreover, the LKB1 loss occurred more frequently in patients harboring co-occurrent alterations within the *KRAS* gene. Conversely, in our study, the LKB1 loss was associated with non-smokers and those without a wood smoke exposure. We attributed these results due to an enrichment of the non-smoker population in our cohort. Non-smokers represented almost 85% of patients, which correlates with the high rate of *EGFR*-mutant patients in approximately 40%; this is in agreement with previous studies performed on Hispanic patients [14,22]. 

LKB1 alterations have been associated with *KRAS* alterations and their prognostic role in this subpopulation. Concurrent loss of LKB1 and *KRAS* mutations has shown enhanced metabolic activity and tumor disease burden reflecting a more aggressive biology than subjects with a single alteration in *KRAS*. In a clinical assessment, *KRAS*-mutant NSCLC patients with LKB1 loss experienced a higher rate of extra thoracic lesions and central nervous system involvement [17]. One limitation in our study was that we were unable to assess other concurrent mutations besides *EGFR* and *ALK* alterations, due to an access barrier to broader genomic panels. Moreover, we cannot assess other mutations of interest, such as *KEAP1* and *STK11,* in order to associate LKB1 mutations with protein expression. 

Limited evidence exists, other than *KRAS* associations with tumor-suppressor genes or oncogenic drivers. Notably, we found that *EGFR* wild-type status was significantly associated with a negative LKB1 expression, and whereas *EGFR* patients had a higher rate of expression [23], we found that almost 30% of our cohort with a LKB1-negative expression harbored an *EGFR* mutation. LKB1 and *KRAS* alterations activate downstream the PI3K-AKT-mTOR and KRAS-RAF-MAPK pathways, and *EGFR* mutations support the idea that these alterations might be mutually exclusive. However, our evidence suggests that both alterations (LKB1 and *EGFR*) may coexist, inducing lung carcinogenesis through modulating the mTOR pathway [23]. Otherwise, LKB1 alterations are considered mechanisms of resistance in EGFR-mutant patients treated with target therapy. Of note, a paired genomic analysis proposed, in a single center study, that an acquired genomic alteration in LKB1 through activation of the PI3K/AKT/mTOR pathway might favor the adenocarcinoma to squamous cell transformation after EGFR-TKIs [24].

One metanalysis, which evaluated eleven studies and 1507 patients with lung cancer, assessed the prognostic role of LKB1 expression. This pooled group showed no association between low or negative LKB1 expression and progression-free survival. In contrast, the OS was significantly worse in those patients with a negative or low LKB1 expression, regardless of the lung cancer subtype (HR 1.67; *p* = 0.024) [11,25]. Of note, the heterogeneity (*I* = 83.5%) was remarkably high in this metanalysis; this was attributed to a wide variety of different cutoff points in the IHC analysis, different clinical stages and distinct time-to-event endpoints. Although LKB1 expression has been predictive of the benefit of antiangiogenic therapy in combination with chemotherapy in other studies, patients with a weak expression or loss of expression did not benefit from adding bevacizumab to chemotherapy [26]. 

In terms of efficacy, our results were in line with previous findings in terms of progression-free and overall survival in the cohort of patients with a negative LKB1 expression. Regardless of the mutational status and administered therapy (TKI or chemotherapy), LKB1 loss was significantly associated with worse survival outcomes. Of note, the frequency of LKB1 loss evaluated by IHC in our population was much higher (65.5%) than in previous reports, even higher than Caucasians. In the multivariate analysis, the LKB1 loss remained a negative factor for PFS and OS after the adjustment for ECOG PS and age; the effect of the EGFR-TKI therapy might explain partially this observation. 

This study has limitations that must be taken into consideration before drawing definitive conclusions. First, even though the used polyclonal antibody is not completely validated in other studies, exhaustive standardization processes, using non-immune positive and negative controls, were carried out to ensure staining quality and specificity of our assays. Secondly, the study was of a retrospective nature, and we could not perform genetic analyses to correlate mutations and the LKB1 expression assessed by IHC; although, some evidence demonstrated that LKB1 mutations were, in the overwhelming majority, predicted to be deleterious for protein function. In a study performed by Skoulidis F. et al., using NGS techniques, the authors identified *STK11/LKB1* alterations, LKB1 expression and its correlation with inferior clinical outcomes and resistance to PD-1 blockades [6]. Additionally, next-generation sequencing platforms are not universally available in LATAM, which reflects daily clinical practice and limitations in the region to identifying other concurrent alterations besides the classic oncogenic drivers. In addition, our cohort did not reflect the impact of current standard treatments due to an access barrier to immunotherapy (IO monotherapy, IO combinations and IO plus chemotherapy combo) in the NSCLC population without oncogenic drivers, and to third-generation EGFR-TKIs as first-line therapy in the EGFR-mutant population.

In summary, our results suggested a potential prognostic role of LKB1 IHC analysis which warrants further studies, ideally prospective designs to allow control of confounding variables. Considering the lack of prognostic markers in lung cancer and looking for accessible and feasible markers which could provide clinicians with prognostic data, the LKB1 expression might be helpful in contexts like LATAM, which are without broad access to next-generation sequencing. If confirmed in future prospective studies where different treatments are evaluated within LKB1 expression, this protein could be used as an available biomarker upon which we can base critical therapy decisions, thereby improving clinical outcomes. In the process, details and potential handicaps in LKB1 determination by immunohistochemistry need to be addressed, such as the lack of standardization in the cutoff points and the different LKB1 clones employed. 

## 5. Conclusions

Genetic alterations on LKB1, which can lead to a truncated protein, were frequent in this advanced NSCLC cohort and associated with non-smokers and an EGFR wild-type population. Moreover, the LKB1 loss assessed by immunohistochemistry was significantly associated with a worse PFS and OS in first-line therapy, in advanced NSCLC patients. The LKB1 IHC analysis is feasible and represents a potential prognostic biomarker at lower costs than genomic DNA or RNA-based analyses. This strategy warrants prospective evaluations to delineate its potential role in places without access to a comprehensive genomic analysis.

## Figures and Tables

**Table 1:** Baseline characteristics of the entire population and according to LKB1 expression.

Characteristics	Overall Population	LKB1 (+) 37 (33.6)	LKB1 (−) 73 (66.3)	*p*
Gender				
Female:	75 (68.2)	25 (33.3)	50 (66.7)	0.922
Male:	35 (31.8)	12 (34.3)	23 (65.7)	
Age				
<60	47 (42.7)	16 (34.0)	31 (66.0)	0.938
≥60	63 (57.3)	21 (33.3)	42 (66.7)	
Smoking status				
Never	88 (80.0)	24 (27.3)	64 (72.7)	**0.005**
Current or former	22 (20.0)	13 (59.1)	9 (40.9)	
Wood smoke exposure				
No	93 (84.5)	25 (26.9)	68 (73.1)	**<0.001**
Yes	17 (15.5)	12 (70.6)	5 (29.4)	
ECOG				
0–1	99 (90.0)	36 (36.4)	63 (63.6)	0.069
2	11 (10.0)	1 (9.1)	10 (90.9)	
Clinical stage				
IIIB	9 (8.2)	3 (33.3)	6 (66.7)	0.984
IV	101 (91.8)	34 (33.7)	67 (66.3)	
NSCLC subtype				
Non-squamous	103 (93.6)	37 (35.9)	66 (64.1)	0.052
Squamous	7 (6.4)	0 (0)	7 (100)	
Differentiation grade *n* = 95				
Well-differentiated	13 (11.8)	3 (23.1)	10 (76.9)	0.081
Moderately differentiated	39 (35.5)	17 (43.6)	22 (56.4)	
Poorly differentiated	40 (36.4)	15 (37.5)	25 (62.5)	
Other	18 (16.4)	2 (11.1%)	16 (88.9)	
*EGFR* status				
Wild-type	80 (72.7)	16 (22.5)	64 (77.5)	**<0.001**
Mutation	30 (27.3)	21 (70.0)	9 (30.0)	

LKB1: liver kinase B1; ECOG: Eastern Cooperative Oncology Group; NSCLC: non-small-cell lung cancer; *EGFR*: epidermal growth factor receptor. Statistical analysis was performed by a Chi-square (x^2^) test, with a *p* ≤ 0.05 significant value. Statistically significant *p*-values are in bold.

**Table 2:** Univariate and multivariate analysis evaluating main clinical factors for progression-free survival.

Characteristics	HR (95% CI)	*p*	HR (95% CI)	*p*
	Univariate		Multivariate	
ECOG				
0–1	-	-	-	-
2	2.20 (1.13–4.28)	**0.021**	0.98 (0.48–2.02)	0.966
Smoking status				
Never	-	-	-	-
Current or former	1.00 (0.61–1.64)	0.993	3.40 (1.83–6.31)	**<0.001**
Differentiation grade				
Well-differentiated	-	-	-	-
Moderately differentiated	0.86 (0.45–1.67)	0.660	1.06 (1.54–2.08)	0.867
Poorly differentiated	0.97 (0.50–1.87)	0.924	1.59 (0.81–3.15)	0.181
NOS	1.58 (0.75–3.31)	0.228	1.48 (0.68–3.21)	0.320
Clinical stage				
IIIB	0.44 (0.20–0.96)	**0.039**	0.32 (0.45–1.39)	**0.011**
IV	-	-	-	-
EGFR status				
Wild-type	-	-	-	-
Mutation	0.39 (0.24–0.63)	**<0.001**	0.32 (0.17–0.59)	**<0.001**
LKB1 expression				
Negative	-		-	
Positive	0.29 (0.18–0.48)	**<0.001**	0.20 (0.11–0.38)	**<0.001**
Response to 1st line therapy				
No response	-	-	-	-
Partial or complete response	0.52 (0.34–0.77)	**0.001**	0.26 (0.16–0.43)	**<0.001**

ECOG: Eastern Cooperative Oncology Group; NOS: not otherwise specified, EGFR: epidermal growth factor receptor; LKB1: liver kinase B1. Statistically significant *p*-values are in bold.

**Table 3:** Univariate and multivariate analysis evaluating main clinical factors for overall survival.

Characteristics	HR (95% CI)	*p*	HR (95% CI)	*p*
	Univariate		Multivariate	
Age				
<60 years	-		-	
≥60 years	1.56 (1.02–2.40)	0.041	1.63 (1.04–2.57)	0.035
Smoking status				
Never	-		-	
Current or former	0.78 (0.45–1.34)	0.365	1.50 (0.73–3.09)	0.265
ECOG PS				
0–1	-		-	
2	3.27 (1.66–6.44)	**0.001**	1.83 (0.87–3.84)	**0.112**
Differentiation grade				
Well-differentiated	-		-	
Moderately differentiated	2.28 (1.03–5.04)	**0.041**	3.89 (1.55–9.75)	**0.004**
Poorly differentiated	3.11 (1.39–6.94)	**0.006**	3.95 (1.60–9.73)	**0.003**
NOS	2.56 (1.08–6.05)	**0.033**	2.53 (0.95–6.79)	0.064
*EGFR* status				
Wild-type	-		-	
Mutation	0.54 (0.33–0.90)	**0.019**	0.79 (0.45–1.39)	0.411
LKB1 expression				
Negative	-		-	
Positive	0.45 (0.28–0.74)	**0.001**	0.34 (0.18–0.65)	**0.001**
Response to 1st line therapy				
No response	-		-	
Partial or complete response	0.62 (0.41–0.95)	**0.027**	0.54 (0.43–0.84)	**0.006**

ECOG: Eastern Cooperative Oncology Group; NOS: not otherwise specified, *EGFR* epidermal growth factor receptor. Statistically significant *p*-values are in bold.

## Data Availability

The data presented in this study are available on request from the corresponding author.
